# Analysis of the unmet needs of Palestinian advanced cancer patients and their relationship to emotional distress: results from a cross-sectional study

**DOI:** 10.1186/s12904-022-00959-8

**Published:** 2022-05-14

**Authors:** Hammoda Abu-Odah, Alex Molassiotis, Justina Yat Wa Liu

**Affiliations:** 1 School of Nursing, The Hong Kong Polytechnic University, Hung Hom, Hong Kong; 2 Nursing and Health Sciences Department, University College of Applied Sciences (UCAS), Gaza, Palestine

**Keywords:** Advanced cancer, Cross-sectional study, Experience, Unmet needs, Palestine, Palliative care

## Abstract

**Background:**

Although several studies assessing the needs of advanced cancer patients have been conducted globally, most have focused on a specific type of cancer such as lung or breast cancer. The variation across studies has also created difficulties in generalizing the results and applying the findings in other countries. The aim of this study was to provide comprehensive information on the needs of Palestinian advanced cancer patients. The quality of life (QOL), distress levels, depression, anxiety, and spiritual well-being of the patients were also assessed.

**Methods:**

A hospital-based study with a cross-sectional design was conducted on a convenience sample of patients aged 18 or above who had been diagnosed with advanced-stage cancer. The unmet needs of the patients were assessed using the Short form of the Supportive Care Needs Survey (SCNS-SF34). Four instruments were utilized to examine their distress, anxiety, depression, QOL, and spirituality. A modified Supportive Care Framework was adopted to guide the design of this study. Descriptive statistics and hierarchical linear regression were utilized to analyse the data.

**Results:**

Of the 404 cancer patients invited to the study, 379 patients consented to participate and complete the questionnaire. Of them 96.8% stated that they had at least one ‘moderate to high’ level unmet need. The most frequent unmet needs were those in the physical aspects of daily living (Mean 58.94; SD ± 20.93) and psychological (Mean 58.84; SD ± 19.49) domains. Most of the patients (91%) were physically ill and reported experiencing physical symptoms. About 78.1% had a high level of distress. Almost 90% reported signs of depression and anxiety. Although they felt that their spiritual well-being was good, their QOL was poor. Hierarchical linear regression analyses confirmed that educational level, age, gender, marital status, cancer stage, cancer type, physical symptoms, depression, anxiety, distress, QOL, and spirituality were independently associated with unmet supportive care needs.

**Conclusion:**

Palestinian advanced cancer patients exhibit a significantly higher prevalence of unmet needs than those in other countries, indicating a need to develop a palliative care programme within the healthcare system. They have a great need for physical, emotional/psychosocial, self-management and other services, which should be made available to them, particularly in the routine delivery of cancer care.

**Supplementary Information:**

The online version contains supplementary material available at 10.1186/s12904-022-00959-8.

## Background

Caring for advanced cancer patients requires continuous follow-ups to ensure that their needs are met and their life is improved [1]. Such patients make up the largest group of patients with life-threatening illnesses in need of support and follow-up [2, 3]. Advanced cancer patients have more unmet needs than those in earlier stages of cancer [4]. They often experience severe pain, distress, and fatigue – all related to the progression of the disease [5, 6]. They are vulnerable and in need of appropriate care in the last stages of their life. Identifying the unmet needs of patients with advanced cancer can help to improve their quality of life (QOL) [7] and reduce hospital admissions [8].

Appropriate care means providing care that meets the needs of patients and alleviates the symptoms that accompany their illness [9]. Needs are defined as ‘the requirement of individuals to enable them to achieve, maintain or restore an acceptable level of social independence or QOL, as defined by particular care agency or authority’ [10]. Healthcare authorities, therefore, should assess the desires of the patients and take them into consideration in order to change current healthcare services [11]. Unmet needs in patients refer to the gap between a patient’s need or expectations for those services and the actual experience of receiving them [12].

The gap between the healthcare services delivered to patients and their expectations of such services can increase the burden on healthcare systems, lead to a surge in healthcare expenditures, and result in harmful effects [13]. Thus, identifying the needs of patients is the first step that needs to be taken to enhance the services that are provided to them [14]. These include pain and symptom management along the trajectory of their disease, and the extension of physical, psychological/emotional, and spiritual assistance [15]. These needs are categorized under the term ‘palliative care’ (PC) [16].

Numerous studies have been conducted to assess the unmet needs of advanced cancer patients. Two recent systematic reviews have focused on such patients; the first, conducted by Moghaddam et al. [17], identified informational, psychological, and physical needs as the most common unmet needs. The second, conducted by Wang et al. [15], showed the main unmet needs as consisting of psychological, physical, healthcare service-related, and informational needs. Other studies have been undertaken with the same aim in mind. For example, in most Western countries psychological needs were identified as needs that frequently went unmet among cancer patients [18]. In Asian countries, Chinese, Japanese, and Korean studies found that the common unmet needs were due to a lack of information provided by those working in the health system [19, 20]. In Arab countries, Nair et al. [21], in a study conducted in the United Arab Emirates, reported psychological needs were the most frequently unmet need. However, the majority of studies have focused on patients with a specific type of cancer such as lung, colorectal, lymphoma, or breast cancer. Meanwhile, the variations in the findings across different countries has led to difficulties in generalizing the findings.

**Fig. 1:**
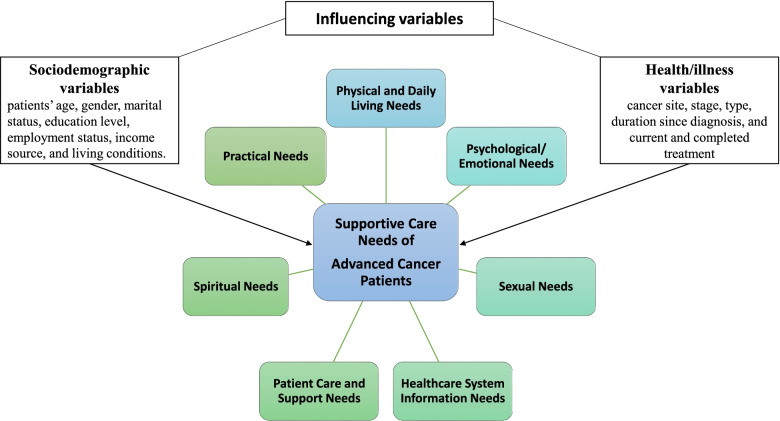
A modified supportive care framework for cancer care [15, 17, 31]

This study focuses on a country (Palestine-Gaza Strip) where the situation in terms of religion, finances, the economy, the healthcare system, and access to services often differs greatly from that in other countries. The Gaza Strip is a narrow band of land populated by 2,018,000 people [22]. Most of the Palestinian people are Muslims who believe in Allah (God) and in the inevitability of death which is Allah’s responsibility [23]. These beliefs and norms help patients cope and accept illness and die in peace [24]. Financial resources in Gaza are scarce, poverty levels are high, financial and administrative coordination are poor, and healthcare resources are in short supply [25]. Cancer currently ranks as the major cause of morbidity and mortality after heart disease and cerebrovascular disease [26]. It is the third leading cause of death (at 14%), with an expected high increase in the cancer burden that will create challenges in the delivery of care to patients that are mostly diagnosed at a late stage [27]. The two Oncology in the Gaza Strip units are housed in inappropriately designed buildings and there are shortages of necessary equipment and supplies [28]. Cancer care whist it is improving in Palestinian hospitals, services like PC, targeted cancer therapies, and bone-marrow transplantation are limited [26, 29]. Furthermore, the shortage of specialist physicians and limited availability of chemotherapy [29, 30]. All of these differences make it difficult to apply the findings from previous studies to the situation in Palestine. This study was conducted to provide comprehensive information on the supportive care needs of advanced cancer patients in Palestine. A modified Supportive Care Framework for Cancer Care (SCNF) was adopted to guide the design of the study and the selection of the outcome variables using an evidence-based approach [31]. The framework, which covers seven domains, has been internationally used in assessments of cancer and stroke care [32]. The framework also includes factors affecting the needs of patients (Fig. 1). The QOL, distress level, depression, anxiety, and spiritual well-being of the patients were also assessed in this study.

## Methods

### Study design

A hospital-based cross-sectional quantitative design was applied in this study.

### Setting

From May 2020 to August 2020, participants were recruited from two hospitals in the Gaza Strip (Al- Shifa Hospital and the European Gaza Hospital) that provide cancer care services to adult patients [33].

### Characteristics of the participants and calculation of sample size

Convenience sampling was adopted to recruit participants for the study. Participants were eligible if they (i) had been diagnosed with stage III or IV cancer, as stated in their medical records; (ii) were aged 18 or above; (iii) were being treated at one of the abovementioned two cancer centres; (iv) had visited the cancer centres as out-patients for follow-up treatment, and (v) were physically able to complete the survey for the study. Patients were excluded if they were unable to complete the survey due to cognitive impairment. These included those suffering from brain tumours and those exhibiting symptoms of cognitive impairment. Patients who met the eligibility criteria were included in the study. Prior to patients’ appointments to visit the clinic, a list of patients’ names who had appointments was printed from the information technology department after getting approval from the hospital director. The printed list was then forwarded to the head of the oncology department to exclude non-eligible patients. The eligible patients on the list were stratified into two groups (Stage III and IV). The patients from each group were chosen.

To minimize the patients’ selection bias, the head nurses were just asked to identify the eligible patients from the list. The list was forwarded to the assigned registered oncology nurses, who were asked to approach the patients and invite the eligible ones to participate in the study after verbal explanations of the study’s aim, benefits and importance. Patients were also provided with a detailed information alongside the questionnaire pack. The nurse asked the patients to take their time to decide if they wanted to participate in this study. The nurse informed patients of the voluntary nature of their participation to the study, their right to withdraw at any time, and that non-participation would not affect the care they receive. Anonymity of their responses was guaranteed. The patients who agreed to participate signed the informed consent form.

The size of the sample was calculated using the Thompson formula: *n = N × p (1-p)/[[N-1 × (d*^*2*^*÷z*^*2*^*)] + p (1-p)]* (*N* = population size, *Z* = confidence level, *d* = error proportion, and *p* = probability) [34]. The result was a sample size of 368 patients (*Z* = 1.96, *d* = 0.05, and *p* = 0.5). This was increased to 404 patients to compensate for non-respondents, as the average non-response rate was reported to be 9.4% in an Italian study [35], and 9.8% in a Chinese study [36].

### Assessment scales

Self-administered questionnaires were adopted to collect data in this study, utilizing multiple survey instruments to minimise differences in the unmet needs across previous studies, to have a comprehensive understanding of advanced cancer patients’ needs and to guide the selection of outcome factors in a more evidence-based approach. To better understand, the theoretical SCNF model was adopted [31] and linked with the findings of previous reviews [15, 17]. This model aims for assessing patients with advanced cancer unmet needs in different areas (physical, spiritual, emotional/ psychological, informational, social, and practical needs and influencing factors) whereas other instruments mainly focus on a particular physio-psychosocial symptom in advanced cancer patients. All instruments utilised in this study have gained permission to use by original authors. The data was collected the months just before the first COVID-19 case was registered in the GS.

### Unmet supportive care needs

The Arabic version of the Supportive Care Needs Survey (SCNS-SF34) was adopted to assess unmet needs [21]. It is a 34-item scale measuring five domains, namely psychological aspects, patient care and support, physical aspects and daily living, health system information, and sexuality [37]. Each item is rated from 1 (no need for help) to 5 (a high need for help). Unmet need items were scored in accordance with the SCNS-SF34 manual [38]. There are two scoring systems proposed by McElduff et al. [38]. The first scoring was based on calculating a Likert summated scale by summing the individual items within a domain. The second scoring creating standardized total scores for each domain to a score out of 100, with a higher score reflecting a higher level of need as perceived by the patients [38]. The second scoring was adopted in our study for comparison of our results with recent studies that utilised the second scoring. The Arabic version of SCNS-SF34 was selected because it is validity and reliability has been confirmed [21].

### Physical symptoms

The Arabic Questionnaire for Symptom Assessment (AQSA) was adopted to assess the presence and quantify the intensity of pain and other common symptoms in cancer patients. The following 11 common symptoms are covered in the AQSA: pain, nausea/vomiting, depression, anxiety, insomnia, dry mouth, tiredness, loss of appetite, confusion, drowsiness, and shortness of breath. Each symptom is rated from 0 (no symptoms) to 10 (worst symptoms). The rating is then classified into one of the following four categories: 0 for no physical symptoms, 1–3 for mild symptoms, 4–6 for moderate symptoms, and 7–10 for severe symptoms. Similar to SCNS-SF34, the Arabic version of AQSA has also been validated [39].

### Emotional/psychological distress

Two instruments were used to assess emotional/psychological distress. The Arabic version of the Distress Thermometer (DT) scale was utilized to identify levels of distress [40]. It is a one-item, self-reported, 11-point visual scale ranging from 0 (no distress) to 10 (high distress). DT also covers 36 problems clustered into five domains. A cutoff score of 4 or more indicates significant distress [40]. The validity of the Arabic version of the DT has been established with best sensitivity (0.70) and specificity (0.63), with cutoff score of 4 [40].

The second instrument, the Hospital Anxiety and Depression Scale (HADS), was adopted to assess the anxiety and depression levels of cancer patients [41]. It is comprised of 14 items under two validated sub-scales: anxiety and depression. The scores in each subscale are computed and determined to fall under one of the following three categories: normal cases (score of 0–7), borderline cases (score of 8–10), and cases (score of 11–21) [42]. The HADS Arabic had high internal consistency with Cronbach’s α coefficient  of 0.83 for anxiety subscale and 0.77 for depression subscale. A strong correction coefficient was showed in the HADS anxiety score (*r* = 0.67) and HADS depression score (*r* = 0.66).

### Quality of life

The Arabic version of the Functional Assessment of Cancer Therapy (FACT-G), containing 27 items, was adopted to assess the QOL of the participants [43]. It covers four domains: physical, social-family, emotional, and functional well-being. The overall scores range from 0 to 108, with higher scores demonstrating better QOL. The FACT-G-Arabic had high internal consistency with Cronbach’s α coefficient was 0.92, with subscales range of 0.73–0.91.

### Spiritual concerns

The Arabic version of the Functional Assessment of Chronic Illness Therapy—Spiritual Well-Being Scale (FACIT-Sp) was utilized to assess spiritual well-being [44]. It is comprised of 12 items distributed over the two sub-domains of peace/meaning and faith. Scores range from 0 to 48, with higher scores indicating greater spiritual well-being. The Cronbach’s α coefficient for the FACIT-Sp Arabic was 0.83.

### Sociodemographic and treatment characteristics

Sociodemographic variables included the patients’ age, gender, marital status, level of education, employment status, source of income, and living conditions. Health/illness variables included cancer site, stage, type, duration since diagnosis, and current and completed treatments (chemotherapy, surgery, radiotherapy, and others).

### Statistical methods

The Statistical Package for the Social Science (SPSS) software version 25 was used to enter and analyse data. Missing data were replaced with multiple imputations. Descriptive statistics were utilized to summarize the sociodemographic and clinical characteristics of the participants as well as all instruments (supportive care needs, physical symptoms, QOL, spirituality, etc.) and their domains. A hierarchical linear regression analysis was employed to test the relationship between the independent variables (distress, anxiety, depression, QOL, and spiritual well-being) and the continuous dependent variable (level of unmet supportive care needs) and to identify a useful subset of possible predictors. Patients’ characteristics information was entered at Model 1 as the predictor for controlling purposes. Quality of life and spirituality variables were entered as Model 2 predictor, while physical symptoms, depression, anxiety, and distress were entered as the Model 3 predictor. All statistical tests were two-tailed, and *p* values of less than 0.05 were treated as significant.

### Ethical considerations

Each patient was provided with a full explanation of the significance and benefits of this study prior to his/her decision to participate in it, and was informed that participation was voluntary. The Ethical Review Committee of The Hong Kong Polytechnic University approved this study (reference number: HSEARS20200414006). Administrative approval was also obtained from the Palestinian Ministry of Health-Gaza (reference number: 476303).

## Results

### Characteristics of the participants

Of the 404 patients who were approached, 379 agreed to take part in this study, for a response rate of 93.8%. The majority of participants were male (*n* = 193, 50.9%) and married (*n* = 316, 83.4%). More than half (199, 52.5%) were above the age of 50, and the mean age was 50.13 ± 14.8 years. Regarding clinical characteristics, half of the study participants were identified as having stage IV cancer. The most common diagnoses were female breast (*n* = 83, 21.8%) and colon (*n* = 58, 15.3%) cancer. The vast majority of participants (*n* = 307, 81.0%) had undergone chemotherapy. Details of the characteristics of the patients are presented in Table 1.

**Table 1:** Characteristics of the participants (*N* = 379)

Socio-demographic variables	Number (%)	Clinical-related variables	Number (%)
**Age Mean (+SD)**	50.13 (14.8)	**Diagnosis/type**	
< 40 years	91 (24.0%)	Breast	83 (21.8%)
40–49 years	89 (23.5%)	Colon	58 (15.3%)
≥ 50 years	199 (52.5%)	Lung	34 (9.0%)
**Gender**		Bone	28 (7.4%)
Male	193 (50.9%)	Prostate	20 (5.3%)
Female	186 (49.1%)	Bladder	12 (3.2%)
**Marital status**		Thyroid	27 (7.1%)
Married	316 (83.4)	Lymphoid	26 (6.9%)
Not married^a^	63 (16.6%)	Brain and neck	25 (6.6%)
**Education**		Stomach	17 (4.5%)
Primary and less	51 (13.5%)	Other	49 (12.9%)
Secondary	243 (64.1%)	**Grade**	
University	85 (22.4%)	III	186 (49.1%)
**Working status**		IV	193 (50.9%)
None	177 (46.7%)	**Duration since diagnosis**	
Employee	102 (26.9%)	Within the last month	23 (6.1%)
Homemaker	100 (26.4%)	1–12 months ago,	136 (35.9%)
**Monthly Income (USD) (*N*** **= 359)**		Over 1 year-3 years ago	129 (34.0%)
Less than 250 USD	249 (69.4%)	Over 3 years ago	91 (24.0%)
More than 250 USD	110 (30.6%)	**Current treatment**	
**Residency/Living conditions**		Chemotherapy	307 (81.0%)
Urban/city	248 (65.4%)	Radiation	27 (7.1%)
Rural	44 (11.6%)	Surgical	16 (4.2%)
Camp	87 (23.0%)	Bone transplantation	2 (0.5%)
		Other	27 (7.1%)

*SD* Standard deviation, *USD* United States Dollar

^a^Includes those who are single, widowed, or divorced

^b^Missing data 5.3%

### Unmet needs of advanced cancer patients

The participants in the study reported having a variety of unmet needs. Four out of five SCNS-SF 34 domains had a score of higher than 50 (Table 2). The highest unmet needs were in the physical /daily living (58.94 ± 20.93; t = − 1.141) and psychological domains (58.84 ± 19.49; t = − 1.192). A total of 96.8% of the patients stated that they had at least one unmet need of a ‘moderate to high’ level. For each of the SCNS-SF 34 items, the percentage of participants who indicated that they had unmet needs ranged from 26.9 to 58.6% (Table 3). The most frequent unmet needs were in the domain of physical/daily living, followed by the psychological domain. Six of the top ten ranked unmet needs were from the psychological domain. The top two items were: ‘Worry that the results of treatment are beyond your control’ (58.6%), and ‘Fears about cancer spreading’ (57.5%). In the physical/daily living domain, four out of the five items ranked among the top ten unmet needs. The top items were: ‘Lack of energy/tiredness’ (54.1%), and ‘Not being able to do the things you used to do’ (53.6%).

**Table 2:** Mean scores for supportive care needs domains

Rank	SCNS SF-34 domain	Mean (±SD)	t-statistic
1	Physical and daily living (range, 0–100)	58.94 (20.93)	−1.141
2	Psychological (range, 0–100)	58.84 (19.49)	−1.192
3	Patient care and support (range, 0–100)	54.17 (21.64)	−5.233
4	Health systems and information (range, 0–100)	51.01 (18.34)	−9.500
5	Sexuality (range, 0–100)	44.05 (26.40)	−11.573

SCNS-SF-34 = Supportive care needs survey short form 34; SD = Standard deviation, t = One sample t test

**Table 3:** Most frequently reported items in the supportive care needs survey

Rank	SCNS-SF- Item	Moderate or high Number (%)	Domain
1	Worry that the results of treatment are beyond your control	222 (58.6)	Psychological
2	Fears about the cancer spreading	218 (57.5)	Psychological
3	Feelings of sadness	209 (55.1)	Psychological
4	Uncertainty about the future	209 (55.1)	Psychological
5	Lack of energy/tiredness	205 (54.1)	Physical and daily living
6	Not being able to do the things you used to do	203 (53.6)	Physical and daily living
7	Feeling down or depressed	191 (50.4)	Psychological
8	Anxiety	186 (49.1)	Psychological
9	Feeling unwell a lot of the time	185 (48.8)	Physical and daily living
10	Pain	174 (45.9)	Physical and daily living

SCNS-SF-34 = Supportive care needs survey short form 34

### Symptoms

The findings revealed that the vast majority of the participants (*n* = 346, 91%) were physically ill and had experienced physical symptoms (49.6% moderate; 41.4% severe). Each item under the domain of physical symptoms was reported to have been experienced by 69.9–95.0% of the participants. Fatigue was the most common symptom (*n* = 360, 95.0%), followed by anxiety (*n* = 346, 91.3%) and pain (*n* = 331, 87.3%) (Table 4).

**Table 4:** Ranking of the intensity of most frequently reported symptoms

Rank	AQSA items	Moderate or severe Number (%)
1	Fatigue/Tiredness	360 (95.0%)
3	Anxiety	346 (91.3%)
2	Pain	331 (87.3%)
4	Depression	318 (83.9%)
5	Loss of appetite	315 (83.1%)
6	Confusion	301 (79.4%)
8	Drowsiness	299 (78.9%)
7	Insomnia	298 (78.6%)
9	Nausea/Vomiting	274 (72.3%)
10	Dry mouth	266 (70.7%)
11	Shortness of breath	265 (69.9%)

*AQSA* Arabic Questionnaire for Symptom Assessment

### Emotional/psychological well-being

About 78.1% of the patients had a high level of distress, with the mean DT score being 6.72 ± 2.48. A total of 67 patients (15%) reported distress at the level of 10, indicating extreme distress. The major sources of distress were related to physical (*n* = 373, 98.4%), emotional (*n* = 359, 94.7%), and practical problems (*n* = 346, 91.3%). A list of the problems is presented in Table 5. About 89.5% of advanced cancer patients reported signs of depression (30.9% borderline; 58.6% definitive, mean depression HADS score of 11.17 ± 3.09), while 87.9% of patients reported signs of anxiety (26.4% borderline; 61.5% definitive, mean score of 11.34 ± 3.38).

**Table 5:** Distress Thermometer list of problems and their frequency as reported by participants

List of problems	Have a problem Number (%)	List of problems	Have a problem Number (%)
**Practical problems**		**Physical Problems**	
Child care	213 (60.9%)	Appearance	235 (62.0%)
Housing	237 (62.5%)	Bathing/dressing	218 (57.5%)
Insurance/financial	213 (56.2%)	Breathing	227 (59.9%)
Transportation	220 (58.0%)	Changes in urination	171 (45.1%)
Work/school	182 (48.0%)	Constipation	207 (54.6%)
Treatment decisions	194 (51.2%)	Diarrhea	174 (45.9%)
**Family Problems**		Eating	246 (64.9%)
Dealing with children	194 (51.2%)	Fatigue	262 (69.1%)
Dealing with partner	187 (49.3%)	Feeling swollen	201 (53.0%)
Ability to have children	178 (47.0%)	Fevers	161 (42.5%)
Family health issues	186 (49.1%)	Getting around	216 (57.0%)
**Emotional Problems**		Indigestion	191 (50.4%)
Depression	276 (72.8%)	Memory/concentration	194 (51.2%)
Fears	275 (72.6%)	Mouth sores	177 (46.7%)
Nervousness	281 (74.1%)	Nausea	210 (55.4%)
Sadness	265 (69.9%)	Nose dry/congested	173 (45.6%)
Worry	256 (67.5%)	Pain	248 (65.4%)
Loss of interest in usual activities	275 (72.6%)	Sexual	150 (39.6%)
**Spiritual/religious concerns**	274 (72.3%)	Skin dry/itchy	145 (38.3%)
		Substance use	113 (29.8%)
		Tingling in hands/feet	208 (54.9%)

### Quality of life and spiritual well-being

The total mean score of the FACT-G was 57.7 ± 11.81, which was slightly below the midpoint of 58. About half of the patients (*n* = 186, 49.1%) scored less than 58. The highest scoring FACT-G subscale was that for social/family well-being (10.68 ± 4.12). The lowest scoring subscale was that for physical well-being (10.39 ± 4.76) (Table 6). The mean spiritual well-being score was 31.12 ± 6.22, reflecting good spiritual well-being. The mean score of the meaning/peace subscale was 18.83 ± 14.18, and that of the faith subscale was 12.26 ± 3.48.

**Table 6:** Mean FACT-G and FACIT-Sp scores

Rank	FACT-G domains	Mean (±SD)
1	Functional Well-Being (range 0–28)	15.71 (5.43)
2	Social/Family Well-Being (range 0–28)	20.92 (5.28)
3	Emotional Well-Being (range 0–24)	10.68 (4.12)
4	Physical Well-Being (range 0–28)	10.93 (4.76)
	FACIT-G Total score (range 0–108)	57.72 (11.81)
	**FACIT-Sp domains**	
1	Faith (range 0–16)	12.26 (3.48)
2	Meaning/Peace (0–32)	18.82 (14.18)
	FACIT-Sp Total score (range 0–48)	31.12 (6.22)

*FACT–G* The Functional Assessment of Cancer Therapy – General, *FACIT-Sp *The Functional Assessment of Chronic Illness Therapy—Spiritual Well-Being Scale

### Factors associated with unmet needs for supportive care and domains of specific needs

Hierarchical regression analyses was utilised to predict variables associated with unmet needs (Table 7). Variables with *p* values less than 0.10 in univariate analysis were considered in regression analysis (Supplement Table S1). Findings showed that level of education was significantly predicted physical aspects of daily living in the regression model I, accounting for 6% of the variation in physical aspects of daily living (*R*^2^ = 0.016). When adding the QOL and spirituality variables to the regression (Model II), the change in *R*^2^ was significant (Δ*R*^*2*^ = 0 .143, *p* = 0 .000), explaining 16% of the variation in physical aspects of daily living (*R*^2^ = 0 .159). When physical symptoms, depression, anxiety, and distress were entered in the regression (Model 3), the change in *R*^2^ was significant (Δ*R*^*2*^ = 0 .068, *p* = 0 .0000), accounting for 23% of the variation in physical aspects of daily living (*R*^2^ = 0 .22). In the final regression model, level of education, QOL and spirituality significantly predicted physical aspects of daily living. Whilst physical symptoms, depression and anxiety significantly predicted physical aspects of daily living, the distress variable was not (Table 7).

**Table 7:** Hierarchical linear regression model for factors associated with supportive care needs

Domain		Model 1			Model 2			Model 3		
		β	***P***		β	***P***		β	***P***	
**Physical & daily living**	Education			R^2^ = 0.016 R^2^ change ΔR^2^ = 0.016 F for change in R^2^ ∆F = 16.96**			R^2^ = 0.159 R^2^ change ΔR^2^ = 0.143 F for change in R^2^ ∆F = 182.58**			R^2^ = 0.22 R^2^ change ΔR^2^ = 0.06 F for change in R^2^ ∆F = 47.42**
	Primary and less	0.10*	0.000		0.07**	0.001		0.11*	0.000	
	Secondary	0.14*	0.000		0.11*	0.000		0.11*	0.000	
	University	–	–		–	–		–	–	
	FACT-G				−0.42*	0.000		−0.33*	0.000	
	FACIT-Sp				0.21	0.000		0.18*	0.000	
	AQSA							0.28*	0.000	
	Depression							0.07**	0.001	
	Anxiety							−0.12*	0.000	
	DL							0.01	0.538	
**Psychological**	Diagnosis			R^2^ = 0.044 R^2^ change ΔR^2^ = 0.004 F for change in R^2^ ∆F = 9.812**			R^2^ = 0.255 R^2^ change ΔR^2^ = 0.211 F for change in R^2^ ∆F = 302.0			R^2^ = 0.358 R^2^ change ΔR^2^ = 0.103 F for change in R^2^ ∆F = 85.374
	Breast	−0.04	0.126		0.04	0.078		0.07	0.004	
	Colon	0.04	0.118		0.11	0.000		0.14	0.000	
	Lung	0.54	0.041		0.05	0.025		0.05	0.012	
	Bone	0.11	0.000		0.15	0.000		0.09	0.000	
	Prostate	0.04	0.051		0.04	0.029		0.02	0.171	
	Bladder	0.02	0.208		0.05	0.016		0.03	0.074	
	Thyroid	−0.05	0.855		0.07	0.002		0.08	0.000	
	Lymphoma	− 0.10	0.000		−0.07	0.001		−0.03	0.069	
	Brain and neck	0.09	0.000		0.10	0.000		0.06	0.002	
	Stomach	0.01	0.752		0.02	0.255		0.04	0.039	
	Other	–	–		–	–		–	–	
	FACT-G				−0.52	0.000		−0.28	0.000	
	FACIT-Sp				0.16	0.000		0.11	0.000	
	AQSA							0.22	0.000	
	Depression							0.03	0.104	
	Anxiety							0.18	0.000	
	DL							0.77	0.000	
**Patient care and support**	Marital status			R^2^ = 0.062 R^2^ change ΔR^2^ = 0.062 F for change in R^2^ ∆F = 12.79**			R^2^ = 0.146 R^2^ change ΔR^2^ = 0.084 F for change in R^2^ ∆F = 105.4			R^2^ = 0.187 R^2^ change ΔR^2^ = 0.041 F for change in R^2^ ∆F = 36.01
	Not married^a^	−0.08	0.000		−0.11	0.000		−0.10	0.000	
	Married	–	–		–	–		–	–	
	Diagnosis									
	Breast	−0.07	0.015		−0.02	0.437		−0.01	0.814	
	Colon	−0.04	0.133		−0.01	0.680		−0.01	0.956	
	Lung	0.11	0.000		0.09	0.000		0.10	0.000	
	Bone	−0.05	0.051		−0.25	0.308		−0.05	0.021	
	Prostate	0.07	0.004		0.06	0.004		0.05	0.013	
	Bladder	0.03	0.157		0.02	0.251		0.01	0.663	
	Thyroid	−0.02	0.272		0.01	0.738		0.01	0.463	
	Lymphoma	−0.09	0.000		−0.07	0.002		−0.06	0.005	
	Brain and neck	0.03	0.014		0.03	0.151		0.01	0.965	
	Stomach	−0.08	0.000		−0.09	0.000		−0.08	0.000	
	Other	–	–		–	–		–	–	
	FACT-G				−0.26	0.000		−0.17	0.000	
	FACIT-Sp				− 0.05	0.012		− 0.01	0.000	
	AQSA							0.23	0.000	
	Depression							−0.09	0.000	
	Anxiety							0.03	0.153	
**Health systems and information**	Age			R^2^ = 0.121 R^2^ change ΔR^2^ = 0.121 F for change in R^2^ ∆F = 22.6**			R^2^ = 0.174 R^2^ change ΔR^2^ = 0.053 F for change in R^2^ ∆F = 68.22			R^2^ = 0.241 R^2^ change ΔR^2^ = 0.067 F for change in R^2^ ∆F = 62.93
	< 40 years	0.03	0.084		0.04	0.049		0.03	0.124	
	40–49 years	0.21	0.000		0.01	0.000		0.16	0.000	
	≥50 years	–	–		–	–		–	–	
	Monthly income									
	< 250 USD	0.06	0.004		0.06	0.002		0.06	0.001	
	≥ 250 USD	–	–		–	–		–	–	
	Diagnosis									
	Breast	−0.11	0.000		−0.08	0.004		−0.07	0.012	
	Colon	−0.13	0.000		−0.11	0.000		− 0.11	0.000	
	Lung	−0.01	0.699		−0.02	0.263		−0.02	0.223	
	Bone	0.177	0.000		0.19	0.000		0.18	0.000	
	Prostate	0.05	0.015		0.06	0.009		0.05	0.019	
	Bladder	0.08	0.000		0.06	0.002		0.04	0.032	
	Thyroid	−0.01	0.457		0.002	0.938		0.01	0.433	
	Lymphoma	0.05	0.026		0.06	0.004		0.06	0.004	
	Brain and neck	0.02	0.237		0.03	0.211		−0.01	0.794	
	Stomach	0.02	0.350		0.01	0.470		0.02	0.261	
	Other	–	–		–	–		–	–	
	FACT-G				−0.12	0.000		−0.09	0.001	
	FACIT-Sp				−0.15	0.000		−0.20	0.000	
	AQSA							0.29	0.000	
	Depression							−0.22	0.000	
	Anxiety							−0.01	0.523	
**Sexuality**	Age			R^2^ = 0.082 R^2^ change ΔR^2^ = 0.082 F for change in R^2^ ∆F = 38.66**			R^2^ = 0.143 R^2^ change ΔR^2^ = 0.061 F for change in R^2^ ∆F = 76.61			R^2^ = 0.181 R^2^ change ΔR^2^ = 0.038 F for change in R^2^ ∆F = 33.255
	< 40 years	0.09	0.000		0.09	0.000		0.07	0.001	
	40–49 years	0.12	0.000		0.11	0.000		0.09	0.000	
	≥50 years	–	–		–	–		–	–	
	Gender									
	Male	0.20	0.000		0.16	0.000		0.15	0.000	
	Female	–	–		–	–		–	–	
	Marital status									
	Not married^a^	−0.06	0.002		−0.09	0.000		−0.09	0.000	
	Married	–	–		–	–		–	–	
	Stage									
	III	−0.14	0.000		−0.12	0.000		−0.95	0.000	
	IV	–	–		–	–		–	–	
	FACT-G				−0.04	0.077		0.09	0.001	
	FACIT-Sp				−2.23	0.000		−0.27	0.000	
	AQSA							0.18	0.000	
	Depression							−0.04	0.057	
	Anxiety							0.10	0.000	

*AQSA* Arabic questionnaire for symptom assessment, *CI* Confidence interval, *DT* Distress thermometer, *EWB* Emotional well-being, *FACT-G* Functional Assessment of Cancer Therapy-General, *FACT-Sp* Functional Assessment of Cancer Therapy- Spiritual, *FWB* Functional well-being, *HDSA* Hospital anxiety depression scale, *PWB* Physical well-being, *SCNS-SF-34* Supportive care needs survey short form 34, *SE* Standard error, *SFWB* Social/family well-being

Regarding the psychological well-being domain, a diagnosis of lung, bone, lymphoma, and brain cancer contributed significantly to the regression model I, accounting for 4.4% of the variation in psychological well-being (*R*^2^ = 0.044). Introducing the attachment variables (Model II) accounted for 25% of variation in psychological well-being and this change in R^2^ was significant (Δ*R*^*2*^ = 0 .211, *p* = 0 .000). Adding the attachment variables (Model III) accounted for 36% of the variation in psychological well-being and this change in *R*^2^ also was significant (Δ*R*^*2*^ = 0 .103, *p* = 0 .0000). In the final regression model, a diagnosis of female breast, colon, lung, bone, thyroid, brain, and stomach cancer were significantly predicted psychological well-being of advanced cancer patients. QOL and spirituality also significantly predicted psychological well-being. Whilst physical symptoms, anxiety and distress significantly predicted physical aspects of daily living, the depression variable was not.

About patient care and support aspects, Model I indicated that marital status, female breast, lung, prostate, lymphoma, and stomach cancer contributed significantly to the regression model, accounting for 6% of the variation in these aspects (R2 = 0.062). When adding the QOL and spirituality variables in Model II, the change in *R*^2^ was significant (Δ*R*^*2*^ = 0 .094, *p* = 0.000), explaining 15% of the variation in patient care and support (*R*^2^ = 0 .146). Adding the attachment variables (Model III), the change in *R*^2^ was significant (Δ*R*^*2*^ = 0.041), *p* = 0 .0000, accounting for 19% of the variation in patient care and support aspects (*R*^2^ = 0.18). In the final regression model, marital status, a diagnosis of lung, bone, prostate, lymphoma, and stomach cancer, QOL and spirituality were significantly predicted patient care and support of advanced cancer patients. Whilst physical symptoms and depression significantly predicted patient care and support aspects, the anxiety variable was not.

Concerning the health systems and information aspects, Model I indicated that age, income, and patients diagnosed of female breast, colon, bone, prostate, bladder, and lymphoma cancer contributed significantly to the regression model, accounting for 12% of the variation in this domain (*R*^2^ = 0.121). When adding the QOL and spirituality variables in Model II, the change in *R*^2^ was significant (Δ*R*^*2*^ = 0 .053, *p* = 0.000), explaining 17% of the variation in health systems and information (*R*^2^ = 0 .174). Adding the attachment variables (Model III), the change in *R*^2^ was significant (Δ*R*^*2*^ = 0.067, *p* = 0 .0000), accounting for 24% of the variation in health systems and information aspects (*R*^2^ = 0.241). In the final regression model, patients whose age ranged from 40 to 49 years, those income less 250 USD, and who diagnosed of female breast, colon, bone, prostate, bladder, and lymphoma cancer were significantly predicted health systems and information unmet needs. QOL and spirituality also significantly predicted health systems and information aspects. Whilst physical symptoms and depression significantly predicted health systems and information unmet needs, the anxiety variable was not.

Concerning the sexual domain, Model I showed that age, gender, marital status, and cancer stage were independently associated with sexual unmet needs, accounting for 8% of the variation in sexual unmet needs (*R*^2^ = 0.082). When adding the QOL and spirituality variables in Model II, the change in *R*^2^ was significant (Δ*R*^*2*^ = 0 .061, *p* = 0.000), explaining 14% of the variation in sexual aspects (*R*^2^ = 0 .143); spirituality significantly predicted sexual aspects. Adding the attachment variables (Model III), the change in *R*^2^ was significant (Δ*R*^*2*^ = 0.038), *p* = 0 .0000, accounting for 18% of the variation in sexual aspects (*R*^2^ = 0.181). In the final regression model, age, gender, marital status, and cancer stage were significantly predicted sexual unmet needs. QOL and spirituality also significantly predicted sexual aspects. Whilst physical symptoms and anxiety significantly predicted sexual unmet needs, the depression variable was not.

## Discussion

This is the first study to have carried out in Palestine. It provided comprehensive information on the needs of advanced cancer patients. A high level of unmet care needs was observed among such patients. Most experienced moderate to severe physical symptoms. Two-thirds screened positive for depression and anxiety. The patients reported a low level of QOL but showed strong spiritual well-being. The multivariable model reported that level of education, age, gender, marital status, cancer stage and type, symptoms, depression, anxiety, distress, QOL, and spirituality were independently associated with unmet supportive care needs.

The Palestinian advanced cancer patients reported significantly higher unmet needs than those observed in earlier studies conducted elsewhere, which ranged between 40 and 72% [18, 19, 21, 45], compared with 96.8% in this study. The highest prevalence was observed in the physical/daily living and psychological domains (58.9 and 58.8% respectively). These results match those identified in earlier studies conducted in Arab and Islamic studies that share religions and cultures. For instance, in the United Arab Emirates, psychological needs were the most frequently unmet need [21]. Among Jordanian people, the most unmet needs were psychological needs [46]. In Indonesia, the Islamic country, physical and psychological needs were also reported as the most frequently unmet needs [47]. In contrast, in Asian countries, Chinese, Japanese, and Korean studies reported that the common unmet needs were due to a lack of information provided by those working in the health system [19, 20, 45], which is inconsistent with the findings of this study. The differences in supportive care needs across countries might be attributed to cultural issues [48, 49]. The significantly higher prevalence of unmet needs in Palestinian patients might be related to the services that are provided to cancer patients in Gaza cancer centres, which are not yet well-prepared and equipped to deliver advanced cancer services such as PC and targeted cancer therapies [27]. Furthermore, the Palestine context might play a role in this high prevalence of unmet needs, as Palestine faces several challenges such as scarce financial resources, high levels of poverty and unemployment, limited infrastructure, and political divisions [50, 51]. As reported in this study, the majority of patients (69.4%) had a low monthly income, which was an obstacle to pursuing advanced and follow-up treatments outside of Gaza [52]. These challenges are further aggravated by the frequent closure of borders and ongoing sieges [53]. These explanations may help us understand why unmet care needs were more common among cancer patients in Gaza. Particular attention must be paid to the patient’s needs. This requires incorporating psychological components of care within the routine cancer delivery should be implemented.

The findings indicate that fatigue (95%), anxiety (91.3%), and pain (87.3%) were the main causes of physical distress. These results matched those conducted in Egypt and Jordan that reported fatigue as the freqent main symptoms among cancer patients [54, 55]. The results also show that nervousness, depression, and fear were the predictive factors of emotional/psychological distress. Unlike in other studies, fatigue, pain, and loss of appetite were the most common symptoms of distress in the patients in this study [40]. Fatigue and pain (physical problems) also ranked among the top 10 predictive factors for distress. Although previous studies corresponded to what this study has reported, the prevalence of physical and emotional/psychological distress is still very high in Palestinian patients. This can be attributed to the unavailability of psychological care in the PC services offered within the Gazan healthcare system [25]. A shortage of cancer healthcare experts in the Gaza Strip [27] and insufficient knowledge and training in the delivery of comprehensive care on the part of healthcare professionals might be another explanation for the high prevalence of physical and emotional/psychological symptoms uncovered in this study. Therefore, measuring physical and emotional/psychological symptoms would be important to enhancing cancer services. The high levels of distress indicate that the priority in developing a PC programme should be on devising treatment strategies to reduce the burden of symptoms. Integrating regular screening as a part of routine cancer care is a practice that is recommended globally for standardizing good care [56]. It is a practice that should be adopted in Palestine. A significant emphasis should be placed on teaching and training healthcare professionals ways of managing the physical and psychological symptoms of patients. This should be done in both service areas and universities. Patients and their families should also be empowered by being instructed on how to manage their health. Therefore, educational programmes should be designed to meet the needs of patients, and PC courses should be introduced in the curriculum of schools of health. High level of distress guides on the development of a PC program. A new guideline should also be developed to address the high distress levels among Palestine patients. Frontline healthcare professionals should be alerted and trained to not only focus on managing patients’ physical symptoms but also be aware the psychological symptoms. Regular screening of all patients with advanced cancer for psychological symptoms is recommended for standardizing good care and alleviating and minimising the stress which impacts on patient’s life and continues good health habits [57]. It should integrate screening as a part of routine cancer care in the Gaza Strip.

Although no spiritual care services are available in the Palestinian healthcare system, the patients were observed to have robust spiritual well-being. This finding is congruent with studies that have been conducted in Jordan and Iran that reported high scores in spiritual well-being among cancer patients [58, 59]. Robust spiritual well-being might be attributed to the Islamic religion and beliefs, as all of the patients were Muslims who believe in God/Allah and in the inevitability of death, which is Allah’s responsibility. Religion and spiritual beliefs have been found necessary for advanced cancer patients due to the confrontation with death [60]. Muslims believe that no one except for Allah can stop or avoid death and illness. They believe in life after death and in eternal life, and agree that life on earth is temporary and could end at any time [61]. After death, every person will be either rewarded or punished in the hereafter [61]. These beliefs and norms help patients to cope, accept their illness, and die in peace [62]. Muslims also believe that tolerating pain is a test of faith and reflects the degree to which patients are connected with Allah [63]. Patients who are able to bear the pain will end by earning a place in Paradise [63].

The majority of advanced cancer patients in this study reported a low level of QOL (mean 57.7 ± SD11.81). This is in line with studies conducted in Palestine that reported low QOL scores (ranging between 41.8 and 49.9%) among advanced cancer patients [64–67]. Slight variations in scores can be attributed to the use of different study instruments. However, these results are not consistent with those of studies conducted in the West [68, 69] and in Arab (Jordan) countries [54, 58]. For example, Al-Natour et al. [58] revealed that the total QOL score among Jordanian cancer patients was 79.86%, compared with 57.7% in this study. This considerable difference across studies may be related to the healthcare systems in Jordan and in Western countries, which are well prepared to deliver PC services and are staffed with well-educated and trained healthcare professionals. It also may be related to the high level of unmet needs, distress, and severity of the symptoms experienced by the participants in our study, which negatively influenced their QOL. To address all of these negative consequences and improve their QOL, therefore, it is recommended that a comprehensive PC programme be integrated within the current healthcare system [70]. The early integration of PC into oncological care can improve patient outcomes, including their QOL [71].

Concerning sociodemographic variables, younger and married male patients were found to have more unmet informational and sexual needs. These findings match those reported in previous studies [19]. Younger patients may ask for more information about their health and the progression of their disease, as well as about their body image, compared with older patients. Other studies have shown that female patients have more needs in the psychological domain [45]. Patterns of unmet needs may differ across cultures and healthcare services that are provided [49]. In Palestine, a low level of unmet needs among females might be attributed to the culture of Arab societies, where females are supported through the expression of empathy and solidarity when they are experiencing severe issues with their health. Or it might be attributed to the conservativeness of a Muslim society and the resulting reluctance and unwillingness of patients to discuss sexual concerns. It is crucial to shed light into this vital area and to find appropriate ways for delivering sexual information, considering patients emotions and feelings. Addressing routine sexual issues are a part of cancer services may overcome cultural barriers and ensuring that this sensitive aspect of patients’ care is met.

For health/illness-related aspects, the results showed that patients with stage IV cancer reported having a higher level of unmet needs, which is consistent with the results of a previous study [4]. Patients who were diagnosed with bone and lymphatic cancers also reported a higher level of unmet needs, specifically related to informational issues. Several studies have reported a significant relationship between cancer site and unmet needs [72, 73]. In these studies, higher levels of unmet needs were reported among patients who had been diagnosed with prostate and lung cancer, which was not the case in this study.

Our results also showed that unmet supportive care needs were independently associated with physical symptoms, psychological distress, and QOL. This finding is consistent with those from previous studies [45, 74]. Understanding the impact of unmet needs on the health and well-being of patients is essential to reducing the severity of their illnesses and increasing survival rates [75]. The findings also revealed that patients with a high level of spiritual well-being were more likely to have a lower level of unmet care needs. When the spiritual needs of patients are not addressed, they are at risk of experiencing emotional/psychological distress [76]. Spiritual care, therefore, should be integrated and matched with the needs of the patients.

### Strengths and limitations

The recruitment of a relatively large and representative sample of patients from the two leading cancer centres in the Gaza Strip has enhanced the generalizability of the results. The different scales that were used in this study all produced the same ranking of problems, an indication that the results of the study can be considered reliable and consistent. The length of the survey instruments used in this study did not affect the willingness of patients to participate in it compared with other studies. Despite the strengths of this study, there were also several limitations. Although it was important to adopt a self-administered questionnaire that allows patients to freely answer the questions, specific types of bias might have resulted. Questions about sexuality may have led to an informational bias due to cultural sensitivities over the subject of sex, posing barriers to open discussions on this subject with patients. Furthermore, adopting the approach of convenience sampling in recruiting participants may have affected the generalizability of the results of the study.

## Conclusion

The advanced cancer patients in this study conducted in Palestine exhibited a significantly higher prevalence of unmet needs, distress, anxiety, and depression than patients in studies previously conducted in other countries. The high prevalence supports the argument that there is a need to develop a PC programme within the healthcare system. Patients have a greater need for different type of services, including physical, emotional/psychosocial, and self-management services, which should be offered within the healthcare system, particularly within the routine delivery of cancer care.

## Supplementary Information

**Additional file 1.**

## Data Availability

The datasets used and/or analysed during the current study are available from the corresponding author on reasonable request.

## Abbreviations

ANOVA: One-way analysis of variance
AQSA: Arabic questionnaire for symptom assessment
DT: Distress Thermometer
FACT-G: The Functional Assessment of Cancer Therapy
FACIT-Sp: The Functional Assessment of Chronic Illness Therapy—Spiritual Well-Being Scale
HADS: Hospital Anxiety and Depression Scale
PC: Palliative care
QOL: Quality of life
SCNF: Supportive Care Framework for Cancer Care
SCNS-SF34: The Supportive Care Needs Survey
SD: standard deviation
SPSS: Statistical Package for the Social Sciences
