# Supplementary material for: Analysis of the unmet needs of Palestinian advanced cancer patients and their relationship to emotional distress: results from a cross-sectional study
Source: BMC Palliat Care. 2022 May 14;21:72. doi: 10.1186/s12904-022-00959-8 (PMC9106510; doi:10.1186/s12904-022-00959-8)
Supplement: Supplementary file 1 — Additional file 1. [file 12904_2022_959_MOESM1_ESM.docx]

**Supplement Table S1: Univariate analysis of socio-demographic and clinical factors that influence patients' needs**

|  | **Physical & daily living** | | | **Psychological** | | | **Patient care and support** | | | **Health systems and**  **information** | | | **Sexuality** | | |
| --- | --- | --- | --- | --- | --- | --- | --- | --- | --- | --- | --- | --- | --- | --- | --- |
| Variables | **Mean (±SD)** | | ***P*** | **Mean (±SD)** | | ***P*** | **Mean (±SD)** | | ***P*** | **Mean (±SD)** | | ***P*** | **Mean (±SD)** | | ***P*** |
| **Age** |  |  | 0.258 |  |  | 0.138 |  |  | 0.298 |  |  | 0.001 |  |  | 0.076 |
| < 40 years | 59.97 | 21.89 |  | 56.35 | 21.12 |  | 52.12 | 25.94 |  | 51.99 | 20.60 |  | 45.62 | 24.57 |  |
| 40-49 years | 61.65 | 17.72 |  | 62.01 | 17.09 |  | 56.95 | 16.50 |  | 56.50 | 15.57 |  | 48.40 | 26.51 |  |
| ≥50 years | 57.94 | 21.75 |  | 58.56 | 19.63 |  | 53.86 | 21.48 |  | 48.10 | 17.86 |  | 41.39 | 27.04 |  |
| **Gender** |  |  | 0.765 |  |  | 0.637 |  |  | 0.115 |  |  | 0.461 |  |  | 0.000 |
| Male | 58.63 | 20.31 |  | 59.30 | 18.61 |  | 55.90 | 20.88 |  | 51.69 | 17.31 |  | 49.17 | 25.01 |  |
| Female | 59.27 | 21.61 |  | 58.37 | 20.41 |  | 52.37 | 22.31 |  | 50.30 | 19.37 |  | 38.74 | 26.85 |  |
| **Marital status** |  |  | 0.488 |  |  | 0.347 |  |  | 0.037 |  |  | 0.508 |  |  | 0.036 |
| Married | 59.33 | 20.11 |  | 58.38 | 19.03 |  | 55.23 | 22.40 |  | 51.31 | 17.91 |  | 45.43 | 25.69 |  |
| Not-married^a^ | 57.03 | 24.67 |  | 61.15 | 21.75 |  | 48.82 | 21.36 |  | 49.48 | 21.68 |  | 37.16 | 28.82 |  |
| **Education** |  |  | 0.043 |  |  | 0.970 |  |  | 0.505 |  |  | 0.211 |  |  | 0.919 |
| Primary and less | 60.49 | 22.36 |  | 58.64 | 20.115 |  | 50.76 | 20.24 |  | 47.15 | 19.25 |  | 43.10 | 24.49 |  |
| Secondary | 60.30 | 20.19 |  | 58.71 | 19.40 |  | 54.72 | 21.44 |  | 52.05 | 18.87 |  | 44.14 | 26.38 |  |
| University | 54.14 | 21.42 |  | 59.31 | 19.96 |  | 54.64 | 23.07 |  | 50.57 | 16.23 |  | 44.37 | 27.84 |  |
| **Monthly income** |  |  | 0.110 |  |  | 0.953 |  |  | 0.456 |  |  | 0.586 |  |  | 0.000 |
| < 250 USD | 59.96 | 20.35 |  | 59.25 | 19.80 |  | 54.04 | 21.03 |  | 51.43 | 18.47 |  | 41.04 | 26.49 |  |
| ≥ 250 USD | 56.22 | 20.41 |  | 59.13 | 16.59 |  | 55.88 | 21.52 |  | 50.38 | 15.77 |  | 52.84 | 22.92 |  |
| **Stage** |  |  | 0.835 |  |  | 0.608 |  |  | 0.113 |  |  | 0.755 |  |  | 0.003 |
| III | 58.72 | 20.67 |  | 59.36 | 19.14 |  | 52.37 | 20.20 |  | 51.31 | 18.37 |  | 39.92 | 26.69 |  |
| IV | 59.17 | 21.23 |  | 58.33 | 19.87 |  | 55.90 | 22.87 |  | 50.72 | 18.34 |  | 48.03 | 25.65 |  |
| **Diagnosis/type** |  |  | 0.246 |  |  | 0.098 |  |  | 0.020 |  |  | 0.001 |  |  | 0.139 |
| Breast | 56.49 | 18.64 |  | 55.75 | 18.05 |  | 51.02 | 19.79 |  | 46.82 | 16.32 |  | 39.97 | 23.33 |  |
| Colon | 55.63 | 18.24 |  | 59.81 | 17.53 |  | 53.27 | 20.74 |  | 44.25 | 17.44 |  | 44.62 | 27.12 |  |
| Lung | 57.20 | 21.04 |  | 61.16 | 16.90 |  | 64.82 | 21.19 |  | 50.04 | 13.51 |  | 45.34 | 23.13 |  |
| Bone | 61.78 | 22.53 |  | 66.25 | 19.25 |  | 50.89 | 26.56 |  | 63.47 | 23.13 |  | 50.83 | 32.73 |  |
| Prostate | 65.00 | 26.50 |  | 62.22 | 15.38 |  | 63.10 | 24.29 |  | 54.77 | 18.47 |  | 51.25 | 27.91 |  |
| Bladder | 64.16 | 17.16 |  | 60.83 | 18.00 |  | 60.83 | 15.93 |  | 58.52 | 13.07 |  | 60.41 | 23.86 |  |
| Thyroid | 54.59 | 22.27 |  | 57.24 | 19.81 |  | 53.34 | 17.48 |  | 52.34 | 18.55 |  | 40.00 | 22.70 |  |
| Lymphoma | 55.38 | 17.48 |  | 49.57 | 22.04 |  | 47.30 | 21.69 |  | 53.37 | 22.90 |  | 41.66 | 23.15 |  |
| Brain and neck | 66.00 | 19.31 |  | 65.02 | 22.53 |  | 58.20 | 21.20 |  | 55.18 | 16.05 |  | 49.66 | 31.50 |  |
| Stomach | 59.41 | 20.82 |  | 58.52 | 22.58 |  | 46.17 | 19.48 |  | 52.94 | 21.19 |  | 47.54 | 24.78 |  |
| Other | 63.40 | 25.55 |  | 57.96 | 21.99 |  | 54.57 | 23.37 |  | 51.50 | 16.76 |  | 38.02 | 28.31 |  |
| **Duration since diagnosis** |  |  | 0.568 |  |  | 0.540 |  |  | 0.935 |  |  | 0.144 |  |  | 0.226 |
| Within last year | 58.22 | 20.44 |  | 59.57 | 20.55 |  | 54.06 | 21.66 |  | 52.63 | 17.65 |  | 42.11 | 26.79 |  |
| Over 1 year ago | 59.47 | 21.31 |  | 58.31 | 18.73 |  | 54.25 | 21.67 |  | 49.83 | 18.76 |  | 45.46 | 26.07 |  |
| **Current treatment** |  |  | 0.737 |  |  | 0.248 |  |  | 0.141 |  |  | 0.113 |  |  | 0.203 |
| Chemotherapy | 59.32 | 19.50 |  | 59.03 | 18.84 |  | 55.30 | 21.55 |  | 52.08 | 17.76 |  | 45.40 | 26.88 |  |
| Radiation | 58.14 | 26.09 |  | 63.83 | 21.23 |  | 51.70 | 20.29 |  | 48.97 | 22.59 |  | 38.14 | 24.24 |  |
| Surgical | 53.37 | 24.34 |  | 55.00 | 25.51 |  | 51.56 | 22.29 |  | 46.02 | 20.85 |  | 38.02 | 25.08 |  |
| Others | 58.79 | 38.11 |  | 54.31 | 20.71 |  | 45.86 | 22.65 |  | 44.34 | 17.68 |  | 38.62 | 22.66 |  |

SD= Standard deviation
